# Neurexin regulates nighttime sleep by modulating synaptic transmission

**DOI:** 10.1038/srep38246

**Published:** 2016-12-01

**Authors:** Huawei Tong, Qian Li, Zi Chao Zhang, Yi Li, Junhai Han

**Affiliations:** 1 Institute of Life Sciences, Key Laboratory of Developmental Genes and Human Diseases, Southeast University, Nanjing 210096, China; 2 Co-Innovation Center of Neuroregeneration, Nantong University, Nantong, JS 226001, China

## Abstract

Neurexins are cell adhesion molecules involved in synaptic formation and synaptic transmission. Mutations in neurexin genes are linked to autism spectrum disorders (ASDs), which are frequently associated with sleep problems. However, the role of neurexin-mediated synaptic transmission in sleep regulation is unclear. Here, we show that lack of the *Drosophila* α-neurexin homolog significantly reduces the quantity and quality of nighttime sleep and impairs sleep homeostasis. We report that neurexin expression in *Drosophila* mushroom body (MB) αβ neurons is essential for nighttime sleep. We demonstrate that reduced nighttime sleep in *neurexin* mutants is due to impaired αβ neuronal output, and show that neurexin functionally couples calcium channels (Cac) to regulate synaptic transmission. Finally, we determine that αβ surface (αβ_s_) neurons release both acetylcholine and short neuropeptide F (sNPF), whereas αβ core (αβ_c_) neurons release sNPF to promote nighttime sleep. Our findings reveal that neurexin regulates nighttime sleep by mediating the synaptic transmission of αβ neurons. This study elucidates the role of synaptic transmission in sleep regulation, and might offer insights into the mechanism of sleep disturbances in patients with autism disorders.

Sleep is an essential and evolutionarily conserved behavior from worm to human^1^,^2^. Sleep disturbances are frequently observed in children with autism spectrum disorders (ASDs), a group of neurodevelopmental disorders^3^. Numerous studies have indicated that sleep disturbances occur in approximately 40–70% of children with ASDs^4^, and primarily include delayed sleep onset, frequent waking during the night, early morning waking, and decreased total sleep time. Sleep traits are largely affected by genetic factors, for example, specific genetic polymorphisms^5^,^6^. The identification of numerous mutations that affect sleep quantity and quality has been crucial to further our understanding of the regulation and functions of sleep^1^,^7^.

Many genetic factors and mutations that contribute to the development of ASDs have been identified. Neurexins and neuroligins are cell adhesion molecules that function in synapse formation^8^,^9^,^10^ and synaptic transmission^11^,^12^. Mutations in neurexin and neuroligin genes have been linked to autism^13^,^14^. Previous studies demonstrated that *neuroligin 1* knockout mice and *neuroligin 4* mutant flies exhibit defective sleep behaviors^15^,^16^. A recent study showed that *neurexin* mutant flies exhibit fragmented sleep, whereas overexpression of neurexin can consolidate nighttime sleep, which might be due to synaptic growth. However, the potential roles and underlying mechanisms of neurexin-mediated synaptic transmission in sleep regulation are largely unknown, and the molecular and neuroanatomical underpinnings of neurexin-regulated sleep are not clear.

*Drosophila* exhibits sleep-like behavior and is a useful model system for genetic studies^17^,^18^. Therefore, *Drosophila* has been used to dissect the molecular regulation and mechanism of sleep^19^,^20^,^21^,^22^. A number of genes^20^,^23^, circuits^21^,^22^, and biological processes^24^ that affect sleep have been identified. *Drosophila* sleep is governed by both circadian and homeostatic regulation^19^,^25^, similarly as in mammals. The large ventral lateral neurons (l-LNvs) mediate light-driven arousal through the release of pigment dispersing factor (PDF), whereas GABAergic inputs to l-LNvs promote sleep. The mushroom bodies (MBs), which contain 7 types of Kenyon cells (KCs), 21 types of output neurons, and 20 types of dopaminergic neurons, also are involved in sleep regulation^21^,^22^,^26^. Previous studies showed that blocking synaptic output from KCs can either increase or decrease sleep depending on the GAL4 driver used^21^,^22^. Convincingly, activation of different types of MB output neurons can either repress or promote sleep^26^,^27^. These results suggest that different KC populations play diverse roles in sleep regulation.

Neurexins are encoded by three genes in mammals, each of which has two promoters to generate α- and β-Neurexins^28^. In *Drosophila*, only a single *α-neurexin* gene (*CG7050*) was identified^9^,^10^. Here, we show that the *Drosophila* homolog of α-neurexin is essential for nighttime sleep. We demonstrate that neurexin controls sleep quantity and sleep homeostasis by mediating the synaptic transmission of αβ neurons. We further reveal that neurexin functionally couples Cac channels to regulate synaptic transmission. Our findings indicate that neurexin-mediated synaptic transmission is crucial for nighttime sleep regulation.

## Results

### *Neurexin* mutant flies exhibit reduced nighttime sleep and impaired sleep homeostasis

**Figure 1:**
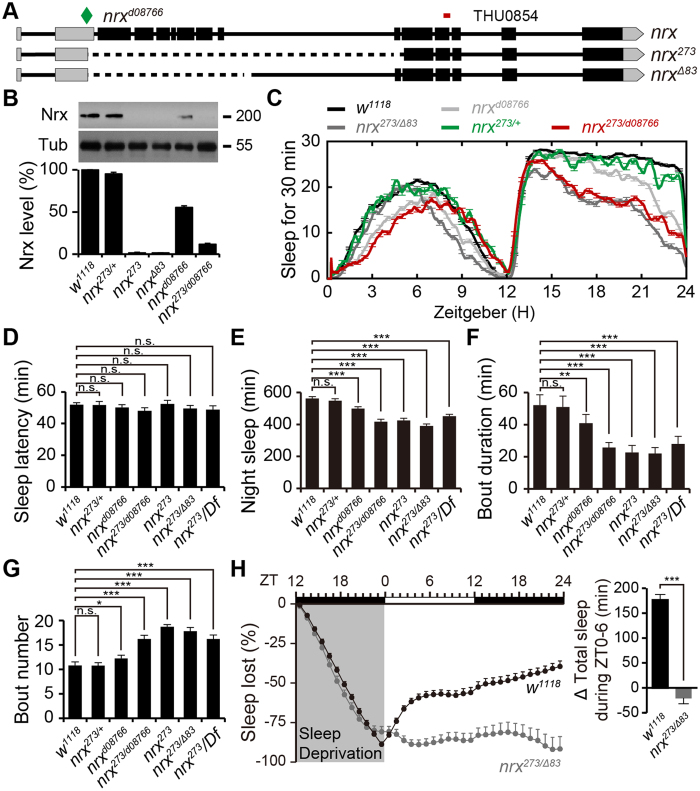
n*eurexin* mutant flies exhibit reduced nighttime sleep. (**A**) Annotated transcription of the *neurexin* gene. The P element XP^d08766^ insertion site is marked with a diamond; the RNAi recognition site is labeled at the top. The deleted regions of *neurexin* mutant alleles (*nrx*^*Δ83*^ and *nrx*^*273*^) are indicated by dashed lines. (**B**) Western blot analysis showing neurexin protein levels in *w*^*1118*^ control and *neurexin* mutant flies. Tubulin was used as a loading control. Quantification of the relative protein level for each genotype is presented in the lower panel. (**C**) Sleep profiles of control *w*^*1118*^ (black, *n* = 308), *nrx*^*d08766*^ (light grey, *n* = 53), *nrx*^*273*^/+ (green, *n* = 32), *nrx*^*273/d08766*^ (red, *n* = 63), and *nrx*^*Δ83***/***273*^ (grey, *n* = 192) flies, which is plotted as a 30 min moving average. (**D–G**) Quantification of sleep onset latency after lights off, total nighttime sleep, average sleep episode length, and number of sleep episodes per night for each genotype. (**H**) After sleep deprivation overnight for 12 h, *nrx*^*Δ83/273*^ mutants regain a significantly lower percentage of their lost sleep than *w*^*1118*^ control flies (mean ± SEM, *n* = 32 flies per group). Quantification of total sleep time during the first 6 h recovery period is presented in the right panel.

To examine the potential roles of neurexin in *Drosophila* sleep, we obtained two null mutant alleles, *nrx*^*Δ83*^ and *nrx*^*273*^, and a hypomorphic allele, *P*{*XP*}*Nrx*^*d08766*^, which contains a transposon inserted in the 5′ untranslated region of the *neurexin* gene (Fig. 1A). After six generations of out-crosses with the *w*^*1118*^ strain, all homozygous *neurexin* mutants were viable. Western blots confirmed the complete loss of neurexin in two *neurexin* null alleles (*nrx*^*Δ83*^ and *nrx*^*273*^), and significant reduction of neurexin in the hypomorphic allele (*P*{*XP*}*Nrx*^*d08766*^) (Fig. 1B). Next, we assessed the sleep behavior of these mutants during 12 h light/dark cycles. *nrx*^*273*^ mutant flies showed normal sleep onset compared with *w*^*1118*^ flies (49.6 ± 1.9 vs. 51.9 ± 1.2 min, Fig. 1C,D). However, compared with *w*^*1118*^ flies, *nrx*^*273*^ mutant flies exhibited a progressive reduction in nighttime sleep (416.3 ± 16.6 vs. 562.5 ± 10.9 min, Fig. 1E) accompanied by shortened episode length (25.7 ± 3.5 vs. 52.1 ± 6.9 min, Fig. 1F) and increased episode number (16.2 ± 0.8 vs. 10.8 ± 0.7, Fig. 1G). These data reflected the poorly consolidated nighttime sleep in *neurexin* mutants. To further eliminate potential genetic background effects, we generated *nrx*^*Δ83/273*^ trans-heterozygotes by combining two out-crossed *neurexin* null mutant alleles, *nrx*^*Δ83*^ and *nrx*^*273*^. Convincingly, similar sleep profiles were observed in *nrx*^*Δ83/273*^ flies and *nrx*^*273*^-deficient flies, in which the mutant allele was combined with *Df(3R)BSC685* (lacking the entire *neurexin* gene) (Fig. 1C and E). These combined observations indicate that neurexin is essential for nighttime sleep.

To further test whether specific levels of neurexin are required for sleep regulation, we compared neurexin protein levels and sleep behavior in *nrx*^*273*^/+ heterozygotes, *P*{*XP*}*nrx*^*d08766*^ mutants, and *p*{*XP*}*nrx*^*d08766/273*^ combination flies. Although *nrx*^*273*^/+ heterozygotes showed a slight reduction in neurexin protein level compared with that of *w*^*1118*^ flies (95.1 ± 2.6%, Fig. 1B), they exhibited normal nighttime sleep (548.0 ± 13.4 vs. 562.5 ± 10.9 min, Fig. 1C and E). *P*{*XP*}*nrx*^*d08766*^ mutants express 53.4 ± 1.8% of the normal amount of neurexin protein expressed by *w*^*1118*^ flies (Fig. 1B), and exhibited reduced nighttime sleep compared with *w*^*1118*^ flies (499 ± 8.2 vs. 562.5 ± 10.9 min, Fig. 1C and 1E). The *p*{*XP*}*nrx*^*d08766/273*^ combination flies contained only 11.2 ± 1.9% of the normal amount of neurexin proteins compared with the levels in *w*^*1118*^ flies, and displayed more severe reductions in nighttime sleep (425 ± 14.3 vs. 562.5 ± 10.9 min, Fig. 1C and E). These combined observations suggest that neurexin mediates nighttime sleep in a dosage-dependent manner.

The timing and amount of sleep are governed by cooperation of circadian and homeostatic mechanisms. We first excluded the possibility that abnormal nighttime sleep in the *neurexin* mutant was due to an altered circadian clock, as *nrx*^*Δ83/273*^ mutants exhibit comparable behavioural periods with those of wild-type flies under constant dark conditions (23.75 ± 0.42 vs. 23.77 ± 0.33 h, Fig. S1). Next, we examined whether reduced sleep in *neurexin* mutants might be due to defective homeostatic regulation. We mechanically deprived flies of sleep for 12 h overnight, and then measured the amount of sleep that was regained during the following 24 h. The *nrx*^*Δ83/273*^ mutants recovered significantly less of the lost sleep than that of *w*^*1118*^ controls (Fig. 1H). These data demonstrate that *neurexin* mutants exhibit defective homeostatic sleep regulation, and suggest that the reduced nighttime sleep in *neurexin* mutants is likely due to alterations in the neural circuits or molecular pathways that govern homeostatic control of sleep.

### Neurexin is expressed in mushroom body neurons and is crucial for nighttime sleep

**Figure 2:**
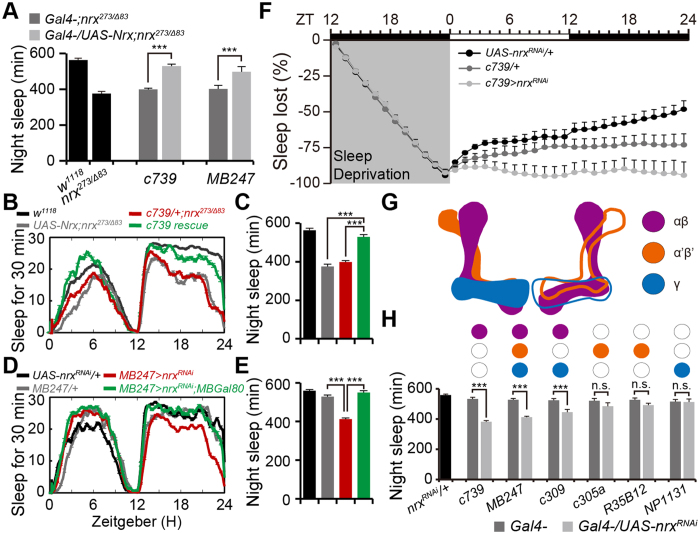
Neurexin expression in mushroom body neurons is essential for nighttime sleep. (**A**) Total nighttime sleep in flies with rescued neurexin expression using anatomically restricted GAL4 drivers. All rescue experiments were performed in the *nrx*^*273/Δ83*^ background; flies carry one copy of the indicated drivers (*n* = 32). (**B,C**) Average sleep profiles for rescued and control flies, with curves plotted as 30 min moving averages (**B**); quantification of total nighttime sleep in rescued and control flies (**C**); *n* = 61. (**D,E**) Average sleep profiles for *MB247-GAL4/UAS-nrx*^*RNAi*^ and control flies, plotted as a 30 min moving average (**D**), and quantification of total nighttime sleep for each genotype (**E**); *n* = 32. (**F**) Cumulative sleep lost during 12 h of sleep deprivation and regained during subsequent recovery for 24 h in *c739-GAL4*/+*;UAS-nrx*^*RNAi*^/+ flies and control flies (*n* = 16). (**G**) Model of the fly MB illustrating the different subsets of intrinsic Kenyon cells (KCs) within the lobes. Purple, αβ lobes; orange, α′β′ lobes; blue, γ lobes. (**H**) Total nighttime sleep in flies with depleted neurexin. Each GAL4 expression pattern is summarized at the top. A single copy of the driver was used for each GAL4 line (*n* = 32).

To investigate how neurexin mediates nighttime sleep, we generated *p[UAS-Neurexin]* transgenic flies and conducted rescue experiments to map the anatomical requirements for neurexin in nighttime sleep using the GAL4/UAS system. Endogenous neurexin is broadly expressed; therefore, nine GAL4 lines that cover neurexin expression patterns or are involved in sleep regulation were selected for the rescue experiments. Both of the *c739-GAL4* and *MB247-GAL4* lines, which label MB neurons^29^, largely recovered nighttime sleep in *neurexin* mutants (Fig. 2A–C). By contrast, other GAL4 lines that label extra-MB regions (e.g., *c316-GAL4, pdf-GAL4, 104y-GAL4*, and *c57-GAL4*) failed to restore impaired nighttime sleep in *neurexin* mutants (Fig. S2A). The glia-specific GAL4 lines (*repo-GAL4* and *gcm-GAL4*) also failed to rescue the impaired sleep (Fig. S2A).

To further validate the anatomical requirements of neurexin for nighttime sleep, we attempted to recapture the sleep deficits in *neurexin* mutants using RNAi against *neurexin* in specific neuronal subpopulations. We found that *MB247-GAL4, c739-GAL4*, and *c309-GAL4* significantly reduced nighttime sleep (Fig. 2D,E and Fig. S2B). The reduced sleep in *MB247-GAL4* was eliminated by combination with MB-GAL80, which suppresses GAL4 activity in MBs (Fig. 2D,E). We failed to significantly repress nighttime sleep using other drivers with more restricted neuronal expression, including *104y-GAL4* in fan-shaped body neurons, *c819-GAL4* and *GMR15C02-GAL4* in ellipsoid body (EB) neurons, *c929-GAL4* and *pdf-GAL4* in large and small ventral lateral (LnV) neurons, and *Ilp-GAL4* in pars intercerebralis (PI) neurons (Fig. S2B). We further investigated whether neurexin expression in MB neurons was required for sleep homeostatic regulation. We found that *c739-GAL4*-mediated ablation of neurexin in MB neurons resulted in impaired homeostatic response to sleep loss (Fig. 2F). These combined observations suggest that neurexin expression in MB neurons is essential for nighttime sleep and homeostatic regulation.

MBs contain Kenyon cells (KCs) and MB output neurons. KCs have been grouped into three classes, γ, α′/β′, and α/β, with each class projecting axons to the eponymous lobe (Fig. 2G)^30^. Fluorescent labeling studies of *MB247-GAL4, c739-GAL4*, and *c309-GAL4* show that all three overlap on MB αβ neurons; therefore, our observations indicate that neurexin expressed in MB αβ neurons is essential for nighttime sleep. To further investigate whether neurexin expressed in MB γ and α′β′ neurons is required for nighttime sleep, we depleted neurexin from these neurons using the GAL4 lines that label γ (*NP1131-GAL4*) and α′β′ (*c305a-GAL4* and *GMR35B12-GAL4*) neurons (Fig. 2H). However, none of these lines significantly repressed nighttime sleep (Fig. 2H). These observations strongly suggest that neurexin expressed in MB αβ neurons is essential for nighttime sleep.

### Neurexin expressed in MB αβ_cs_ neurons promotes nighttime sleep

**Figure 3:**
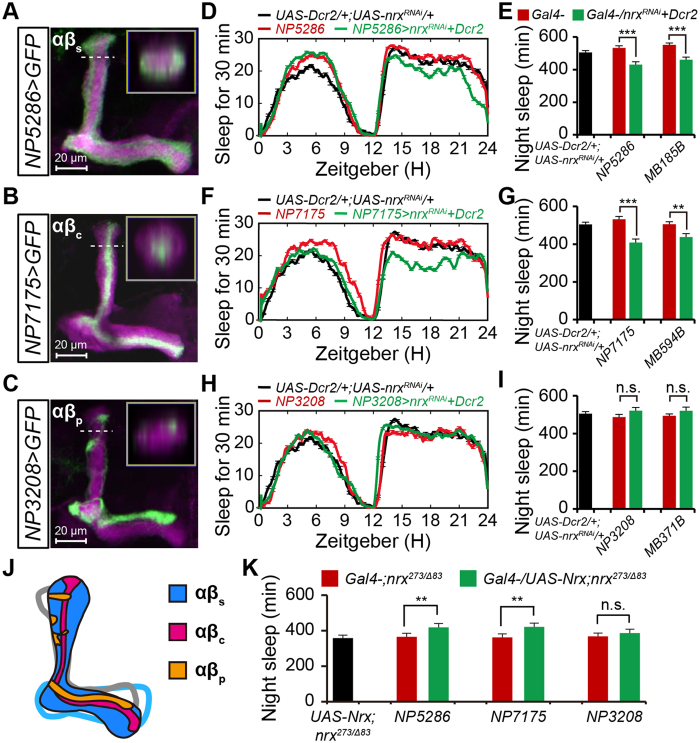
Neurexin functions in αβ_sc_ neurons to promote nighttime sleep. (**A–C**) Projection views of confocal stacks at the level of the right MB lobes from NP5286-αβ_s_ (**A**), NP7175-αβ_c_ (**B**), and NP3208-αβ_p_ (**C**) flies driving mCD8::GFP (green). In all panels, the αβ lobes are labeled with anti-FasII (magenta). The inset shows a horizontal cross-section through the vertical collateral at the level of the dashed line in each panel. Scale bar = 20 μm. (**D,E**) Average sleep profiles for *UAS-Dicer2/NP5286-GAL4;UAS-nrx*^*RNAi*^/+ (*n* = 53) and control (*n* = 32) flies, plotted as a 30 min moving average (**D**), and quantification of total nighttime sleep for each genotype (**E**). Note that Dicer-2 was used to enhance the transgenic RNAi effect in panels D, F, and H. (**F,G**) Average sleep profiles for *NP7175-GAL4/Y;UAS-Dicer2*/+*;UAS-nrx*^*RNAi*^/+ (*n* = 37) and control (*n* = 32) flies, plotted as a 30 min moving average (**F**), and quantification of total nighttime sleep for each genotype (**G**). (**H,I**) Average sleep profiles for *NP3208-GAL4/Y;UAS-Dicer2*/+*;UAS-nrx*^*RNAi*^/+ (*n* = 42) and control (*n* = 32) flies, plotted as a 30 min moving average (**H**), and quantification of total nighttime sleep for each genotype (**I**). (**J**) Model of the MB αβ neurons illustrating three subsets of neurons within the αβ lobes. Purple, αβ_c_ neurons; blue, αβ_s_ neurons; yellow, αβ_p_ neurons. (**K**) Total nighttime sleep in flies with rescued neurexin expression using specific GAL4 drivers for each αβ subset. Rescue experiments were conducted in the *nrx*^*273/Δ83*^ background; flies bear one copy of the indicated drivers (*n* = 16).

MB αβ neurons have been classified into three types according to the parallel axon fibers occupying different layers within the αβ lobes, αβ_c_, αβ_s_, and αβ_p_^26^. To further explore the potential roles of neurexin expressed in each αβ neuron type for nighttime sleep, we obtained three GAL4 lines that label different subsets of αβ neurons. Previous reports showed that *NP7175-GAL4, NP5286-GAL4*, and *NP3208-GAL4* were expressed in αβ_c_, αβ_s_, and αβ_p_ neurons, respectively^29^,^31^. Our confocal projection images confirmed the restricted expression of these lines in each αβ neuron type (Fig. 3A–C). To enhance the transgenic RNAi effects, we overexpressed a component of the RNAi machinery, Dicer2^32^. We depleted neurexin from individual αβ-subset neurons, and then assessed nighttime sleep in these flies. Both *NP5286-GAL4* and *NP7175-GAL4* resulted in reduced nighttime sleep (Fig. 3D–G). By contrast, *NP3208-GAL4* did not significantly affect nighttime sleep (Fig. 3H,I). To further confirm these observations, we obtained a set of validated split-GAL4 lines specific for each αβ subset^26^ and depleted neurexin using these GAL4 lines. Convincingly, both αβ_s_-specific *MB185B-GAL4* and αβ_c_-specific *MB594B-GAL4* dramatically reduced nighttime sleep (Fig. 3E and G), whereas αβ_p_-specific *MB371B-GAL4* flies exhibited normal nighttime sleep (Fig. 3I).

To further validate the role of neurexin in nighttime sleep, we performed rescue experiments by expressing neurexin in individual αβ-subset neurons. Convincingly, expression of neurexin with either *NP5286-GAL4* or *NP7175-GAL4* largely increased nighttime sleep, whereas expression of neurexin with *NP3208-GAL4* had no clear rescue effects on nighttime sleep (Fig. 3J,K). Taken together, these data demonstrate that neurexin functions in αβ_sc_ neurons to promote nighttime sleep.

### Knockdown of neurexin expression in adult flies reduces nighttime sleep

**Figure 4:**
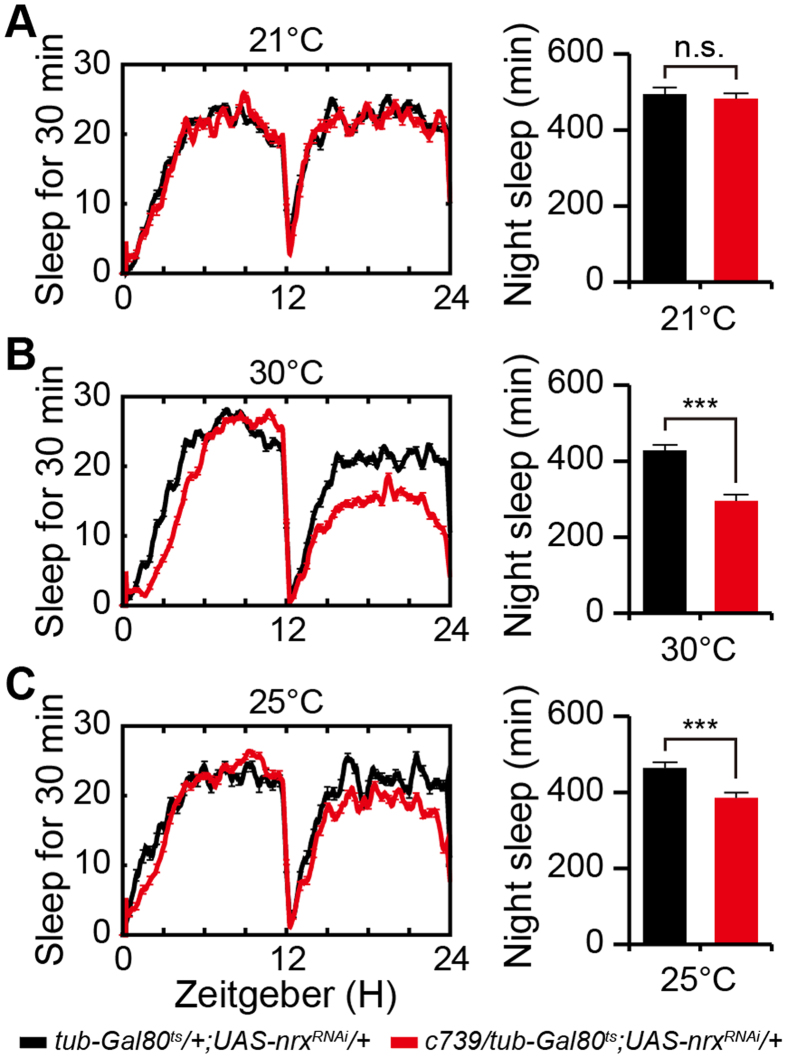
Knockdown of neurexin expression in adult flies reduces nighttime sleep. (**A**–**C**) Sleep profiles for *c739-GAL4/tub-GAL80*^*ts*^*;UAS-nrx*^*RNAi*^/+ (black) and *c739-GAL4/tub-GAL80*^*ts*^ control (red) flies at 21 °C (**A**), 30 °C (**B**), and 25 °C (**C**). Quantification of total nighttime sleep at each temperature is presented in the right panel (*n* = 16).

The abnormal sleep observed in neurexin mutants might be due to developmental defects or impaired synaptic function in adult flies. To distinguish these possibilities, we generated *tubulin-GAL80*^*ts*^/*c739-GAL4;UAS-nrx*^*RNAi*^/+ flies and analyzed their sleep behavior. In these flies, RNAi was suppressed during development by the ubiquitous expression of temperature-sensitive GAL80^ts^, but was selectively induced at adulthood by exposure to 30 °C. Flies were reared at 21 °C during development. Adult flies were entrained for 2 days in light/dark at 21 °C, treated with 30 °C for 3 days to inactivate temperature-sensitive GAL80^ts^, and then returned to 25 °C. Compared with the control flies, *tubulin-GAL80*^*ts*^/*c739-GAL4;UAS-nrx*^*RNAi*^/+ flies exhibited a reduction in nighttime sleep after switching to 30 °C (Fig. 4A,B). Two days after the return to 25 °C, *tubulin-GAL80*^*ts*^/*c739-GAL4;UAS-nrx*^*RNAi*^/+ flies still exhibited reduced nighttime sleep (Fig. 4C). These data indicate that knockdown of neurexin expression in adult flies is sufficient to reduce nighttime sleep.

### Defective nighttime sleep in *neurexin* mutants is due to impaired αβ neuron synaptic transmission

**Figure 5:**
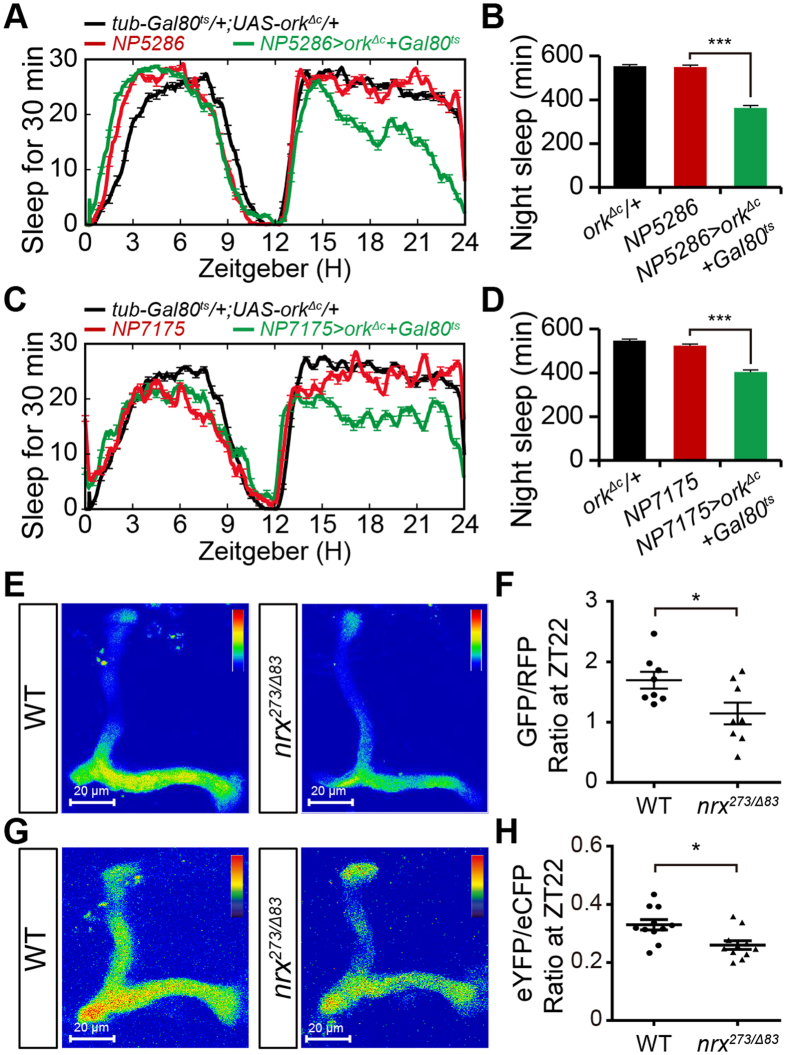
Blocking synaptic output from αβ_sc_ neurons reduces nighttime sleep. (**A,B**) Average sleep profiles for *NP5286/tub-GAL80*^*ts*^*;UAS-Ork1*^*ΔC*^/+ and control flies, plotted as a 30 min moving average (**A**), and quantification of total nighttime sleep in *NP5286/tub-GAL80*^*ts*^*;UAS-Ork1*^*ΔC*^/+ and control flies (**B**) (*n* = 16). For (**A**) and (**C**), all flies were reared at 18 °C throughout development, and adults were entrained at 30 °C for 6 hours every day for 2 days. Then, the flies’ sleep was measured. (**C,D**) Average sleep profiles for *NP7175/Y;tub-GAL80*^*ts*^/+*;UAS-Ork1*^*ΔC*^/+ and control flies, plotted as a 30 min moving average (**C**), and quantification of total nighttime sleep in *NP7175/Y;tub-GAL80*^*ts*^/+*;UAS-Ork1*^*ΔC*^/+ and control flies (**D**) (*n* = 24). (**E**) Representative images of GCaMP6 fluorescence in both *w*^*1118*^ control and *nrx*^*273/Δ83*^ mutant αβ lobes. Images show the sum of fluorescence intensity from multiple layers primarily consisting of αβ lobes at ZT22. (**F**) Quantification of GCaMP6/RFP fluorescence ratios in *w*^*1118*^ control and *nrx*^*273/Δ83*^ mutant αβ neurons at ZT22. RFP fluorescence was used as control (*n* = 8). (**G**) Averaged eYFP/eCFP fluorescence ratio in both *w*^*1118*^ control and *nrx*^*273/Δ83*^ mutant αβ lobes at ZT22. (**H**) Quantification of eYFP/eCFP ratios in *w*^*1118*^ control and *nrx*^*273/Δ83*^ mutant αβ neurons at ZT22 (*n* = 11).

Previous studies showed that blocking the synaptic output of αβ neurons reduced nighttime sleep, whereas hyperpolarization of these neurons has no clear promotion effects on nighttime sleep^22^,^27^. To further clarify the role of each αβ-subset neuron in nighttime sleep, we silenced each subset by expressing a mutant form of the open rectifier potassium channel (dORK^ΔC^)^33^. To exclude the potential roles of dORK^ΔC^ in neural development, its expression was suppressed during development by the ubiquitous expression of temperature-sensitive GAL80^ts^, and then selectively induced in adult flies by exposure to 30 °C^34^. Compared with control flies, ectopic expression of dORK^ΔC^ under the control of either *NP5286-GAL4* or *NP7175-GAL4* significantly reduced sleep (Fig. 5A–D), resembling the defective sleep patterns observed in flies with neurexin selectively depleted from the corresponding αβ-subset neurons. We also expressed the temperature-sensitive cation channel TrpA1 to increase neural excitability and subsequently reinforce αβ neuron synaptic transmission^35^. Consistent with a previous report^36^, activation of TrpA1 by exposure to elevated temperature did not significantly affect nighttime sleep in each αβ-subset neuron (Fig. S3).

These observations suggest that defective nighttime sleep in *neurexin* mutant flies might be due to reduced synaptic transmission of αβ neurons. To compare neural activities, we expressed *GCaMP6.0* in αβ neurons and subsequently performed Ca^2+^ imaging analysis. The αβ neurons were visualized by membrane expression of an RFP-tagged mCD8. Compared with wild-type αβ neurons, *neurexin* mutant αβ neurons showed reduced fluorescence intensity of GCaMP6.0 at ZT22 (Fig. 5E,F). To further validate these observations, we applied the calcium sensor Cameleon 2.1 (Cam2.1), which enables ratiometric measurements of calcium-induced fluorescence resonance transfer (FRET) between enhanced cyan fluorescent protein (eCFP) and enhanced yellow fluorescent protein (eYFP)^37^. Convincingly, compared with wild-type αβ neurons, *neurexin* mutant αβ neurons showed increased eCFP signals and reduced eYFP signals at ZT22 (Fig. 5G,H). These data demonstrate that neural activity and subsequent synaptic transmission are impaired in *neurexin* mutant αβ neurons.

### Neurexin regulates synaptic transmission through functional coupling of Cac channels

**Figure 6:**
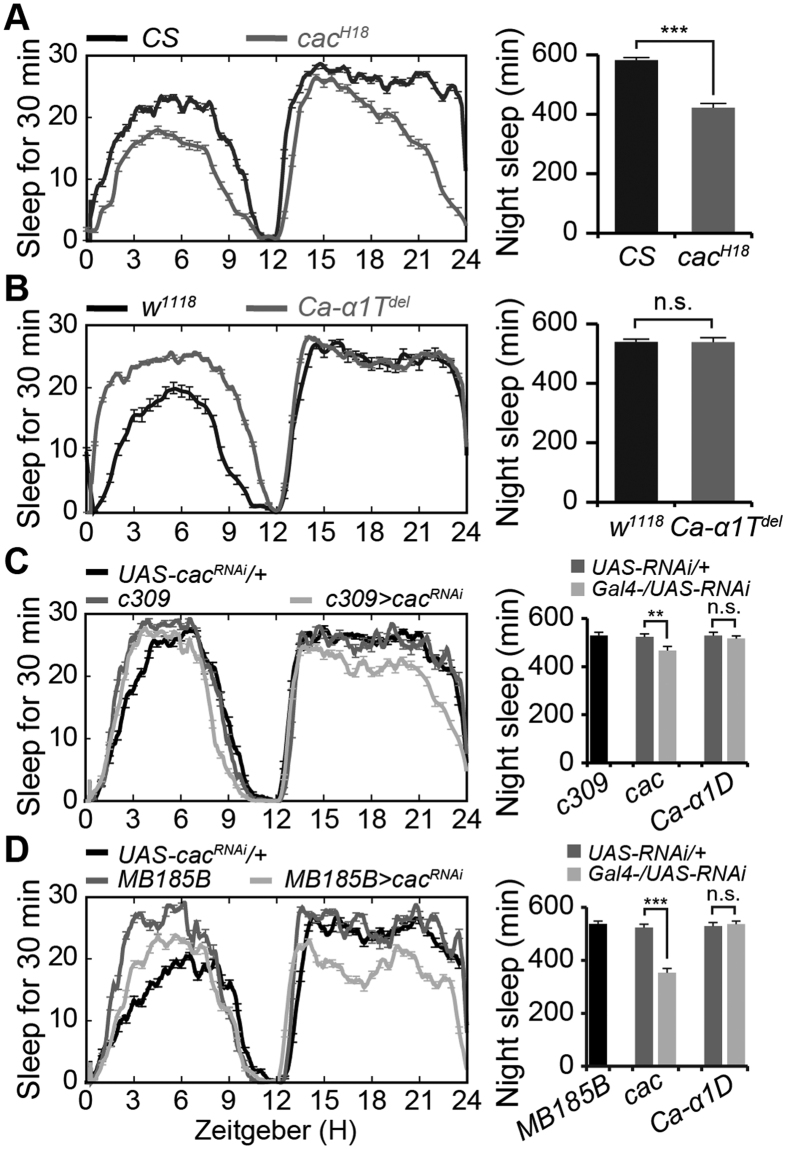
Neurexin functionally couples with calcium channels to mediate synaptic transmission. (**A,B**) Averaged total sleep per night in *cac*^*H18*^ mutants and *Canton-S (CS*) flies (**A**), and in *Ca-α1T*^*del*^ mutants and *w*^*1118*^ control flies (**B**), plotted as a 30-min moving average (*n* = 64). Quantification of total nighttime sleep is presented in the right panel of each figure. Note that *CS* flies were used as the control flies for *cac*^*H18*^ mutants, which were generated in the *CS* background. (**C**) Averaged total sleep per night in *c309-GAL4*/+*;UAS-cac*^*RNAi*^/+ and control flies, plotted as a 30 min moving average (*n* = 32). Quantification of total nighttime sleep in each genotype is presented in the right panel. (**D**) Averaged total sleep per night in *MB185B-GAL4*/+*;UAS-cac*^*RNAi*^/+ and control flies, plotted as a 30 min moving average (*n* = 20). Quantification of total nighttime sleep in each genotype is presented in the right panel.

Previous studies suggest that synaptic transmission may depend on Ca^2+^ entry via voltage-gated Ca^2+^ channels (VGCCs)^38^. We attempted to identify which Ca^2+^ channel was regulated by neurexin in αβ neurons. The *Drosophila* genome contains only three putative homologs of vertebrate voltage-gated Ca^2+^ channels (Ca-*α*1D, Cac, and Ca-*α*1T)^39^. Cacophony channel mutant (*cac*^*H18*^) flies exhibited reduced nighttime sleep compared with that of control flies (422.1 ± 15.3 vs. 582.3 ± 9.4, Fig. 6A), similar to the defective nighttime sleep pattern observed in *neurexin* mutants. Although *Ca-α1T*^*del*^ mutant flies exhibited increased daytime sleep compared with that of control flies (458 ± 10.8 vs. 298 ± 9.7 min), they exhibited normal nighttime sleep (538.4 ± 7.9 vs. 549.7 ± 12.3 min, Fig. 6B), consistent with a previous report^40^. In addition, αβ neuron-specific RNAi against *cac* but not *Ca-α1D* mimicked the defective nighttime sleep in *neurexin* mutant flies (Fig. 6C), and αβ_s_ neuron-specific RNAi against *cac*, but not *Ca-α1D*, recaptured the reduced nighttime sleep (Fig. 6D).

Defective synaptic transmission in *neurexin* mutants could be due to either abnormal Cac channel coupling or altered Cac channel distribution. To distinguish between these possibilities, we compared the levels and distribution of Cac channels in wild-type and mutant Kenyon neurons. Ca^2+^ channels are extremely sensitive to fixation; therefore, we utilized a GFP-tagged Cac transgene (*Cac-GFP*) and captured live images to visualize Cac channels *in vivo*^10^,^41^. Although reduced levels of active zone protein Brp were observed in the mutant α-lobes (Fig. S4A), Cac-GFP levels appeared normal in *neurexin* mutant α-lobes compared with those in controls (Fig. S4B), which was consistent with observations from *neurexin* mutant neuromuscular junctions^10^. Convincingly, N-type Ca^2+^ channels showed normal levels and affinities in several α-neurexin knockout mice^11^. Furthermore, overexpression of Cac channels failed to restore defective sleep in mutant MB neurons (Fig. S4C). Taken together, these results indicate that impaired synaptic transmission in *neurexin* mutants is most likely due to changes in Ca^2+^ channel coupling rather than Ca^2+^ channel distribution.

### The αβ_s_ neurons release both acetylcholine and short neuropeptide F, whereas α β c neurons use sNPF to promote nighttime sleep

**Figure 7:**
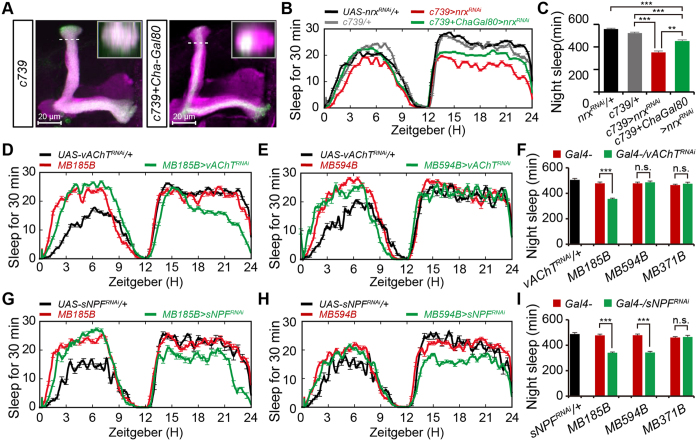
The αβ_s_ neurons release both acetylcholine and short neuropeptide F to promote nighttime sleep, whereas α β c neurons release sNPF to maintain nighttime sleep. (**A**) Expression of c739 in αβ_sp_ neurons is blocked by Cha-GAL80. The inset shows a horizontal cross-section through the vertical collateral at the level of the dashed line in each panel. Scale bar = 20 μm. (**B,C**) Averaged total sleep per night in *c739-GAL4*/+*;UAS-nrx*^*RNAi*^/+ and *c739-*GAL4^*i*^/+*;Cha-GAL80/UAS-nrx*^*RNAi*^ flies, plotted as a 30 min moving average (**B**), and quantification of total nighttime sleep in each genotype (**C**) (*n* = 64). (**D,E**) Averaged total sleep per night in *MB185B-GAL4/UAS-vAChT*^*RNAi*^ and control flies (**D**) (*n* = 64), and in *MB594B-GAL4/UAS-vAChT*^*RNAi*^ and control flies (**E**) (*n* = 24), plotted as a 30 min moving average. (**F**) Quantification of total nighttime sleep in vAChT-RNAi flies and control flies. (**G,H**) Averaged total sleep per night in *MB185B-GAL4/UAS-sNPF*^*RNAi*^ and control flies (**G**) (*n* = 32), and in *MB594B-GAL4/UAS-sNPF*^*RNAi*^ and control flies (**H**) (*n* = 32), plotted as a 30 min moving average. (**I**) Quantification of total nighttime sleep in sNPF^RNAi^ flies and control flies.

Finally, we investigated which neurotransmitter was released by each αβ-subset neuron to promote sleep. Down-regulation of acetylcholine (ACh) signaling in MBs can inhibit sleep^42^; therefore, we first investigated which type of αβ neuron was cholinergic. Cha-GAL80, which suppresses GAL4 activity in cholinergic neurons, successfully repressed the GAL4 activity of *c739-GAL4* in αβ_sp_ but not αβ_c_ subset neurons (Fig. 7A). Compared with the sleep profile of *c739-GAL4*/+ *;UAS-nrx*^*RNAi*^/+ flies, those that also expressed Cha-GAL80 exhibited increased nighttime sleep (452.6 ± 10.7 vs. 351.3 ± 13.1 min, Fig. 7B and C). These observations suggest that αβ_s_ neurons are cholinergic and their ACh signaling promotes nighttime sleep. However, the combination of Cha-GAL80 in *c739-GAL4*/+*;UAS-nrx*^*RNAi*^/+ flies did not fully restore the reduced nighttime sleep (Fig. 7B and C). These observations suggest that some non-cholinergic c739 neurons may promote nighttime sleep, or some cholinergic neurons may be mislabeled by Cha-GAL80.

To further examine whether ACh signaling from αβ_s_ neurons promotes nighttime sleep, we depleted vesicular acetylcholine transporter (vAChT) required for acetylcholine transport and release. Depletion of vAChT in αβ_s_ neurons, but not αβ_c_ and αβ_p_ neurons, resulted in reduced nighttime sleep (Fig. 7D–F), similar to the defective sleep observed in *neurexin* mutant flies. By contrast, down-regulation of other neurotransmitters in αβ_s_ neurons, including glutamate and gamma-aminobutyric acid, did not significantly affect nighttime sleep (Fig. S5A). Taken together, these results demonstrate that αβ_s_ neurons release ACh to promote nighttime sleep.

Neuropeptides are important intercellular signaling molecules in animals, and some genes that encode neuropeptides and their receptors are involved in sleep in *Drosophila*^43^,^44^. Given that down-regulation of all identified neurotransmitters in αβ_c_ neurons still resulted in normal nighttime sleep, we next examined whether αβ_c_ neurons use neuropeptides to maintain sleep by investigating the sleep-promoting roles of four potential neuropeptides known to be expressed in MBs, including FMRFamides (FMRFa), sulfakinins (*X*SK), short neuropeptide F (sNPF), and tachykinin-related peptide (TKRP). The results showed that αβ_s_ neuron-specific depletion of sNPF but not the other neuropeptides caused reduced nighttime sleep (Fig. 7G, and Fig. S5B). In addition, depletion of sNPF in αβ_c_ neurons also reduced nighttime sleep (Fig. 7H and I, Fig. S5C), whereas down-regulation of sNPF in αβ_p_ neurons resulted in normal nighttime sleep (Fig. 7I). These observations indicate that αβ_c_ neurons use sNPF to promote nighttime sleep.

Taken together, our results demonstrate that neurexin promotes nighttime sleep by regulating synaptic transmission. Using *neurexin* mutants, we revealed that αβ_s_ neurons release both ACh and sNPF, whereas αβ_c_ neurons release sNPF to promote nighttime sleep.

## Discussion

Sleep is correlated with neural activity, and numerous mutations that alter neuronal excitability and the balance of inhibitory over excitatory neurotransmission have been shown to significantly affect sleep quantity and quality. In the present study, we showed that *neurexin* mutant flies exhibited reduced and fragmented nighttime sleep. A recent study reported that combinations of hypomorphic *neurexin* alleles led to fragmented sleep, but the total time of night sleep did not differ among these flies^45^. Given that combinations of hypomorphic *neurexin* alleles lead to a partial reduction in *neurexin* mRNA levels^45^, it is reasonable that *neurexin* null mutants exhibit more severe sleep deficits than combinations of hypomorphic alleles. Convincingly, we showed that flies with the hypomorphic *neurexin* allele *P*{*XP*}*nrx*^*d08766*^, which produce 53% of the neurexin level of wild-type flies, exhibited reduced nighttime sleep. We also showed that *neurexin* mutant flies exhibited impaired sleep homeostasis, and identified the anatomical requirements and mechanism of neurexin in nighttime sleep regulation.

The *Drosophila* genome contains only a single *α-neurexin* gene, and immunostaining analysis reveals its ubiquitous expression in nervous system^9^,^10^. Our RNAi knockdown and rescue experiments showed that *α-neurexin* expression in MBs is essential for nighttime sleep, consistent with previous studies showing that MB neurons are involved in sleep regulation^21^,^22^. Our anatomically restricted manipulations of neurexin further revealed that neurexin expressed in αβ_sc_ neurons, but not αβ_p_ neurons, controls nighttime sleep. Given that expression of neurexin in MB neurons does not fully restore defective nighttime sleep in *neurexin* null mutant flies, we suspected that neurexin expressed in other neurons also may function in sleep regulation. We excluded the possibility that neurexin regulated nighttime sleep through several identified sleep-promoting neurons, including fan-shaped body neurons, EB neurons, larger LNv neurons, and PI neurons^46^,^47^. Elucidating the roles of neurexin in other potential sleep-promoting neurons will require additional study.

Neurexins are cell adhesion molecules that function in various neural circuits through mediating synapse formation and synaptic transmission^8^,^48^. A recent report suggested that defective sleep in *neurexin* mutants is not solely due to developmental effects because it can be induced acutely in adults and there is evidence that it functions in synaptic growth^45^. Our study showed that knockdown of neurexin in adult flies reduced nighttime sleep, and neurexin regulated nighttime sleep by modulating synaptic transmission. Our Ca^2+^ imaging analyses revealed that *neurexin* mutant αβ neurons had reduced Ca^2+^ levels compared with those of wild-type neurons, consistent with electrophysiological recordings showing impaired synaptic transmission in *neurexin* mutant neuromuscular junctions^10^. Neurotransmitter release is mediated by the fusion of synaptic vesicles triggered by Ca^2+^, and α-neurexin functionally couples Ca^2+^ channels to the presynaptic machinery to mediate synaptic vesicle exocytosis^11^. In this study, we show that Cac channels are expressed in αβ neurons and mediate synaptic transmission. Therefore, impaired synaptic transmission is correlated with reduced nighttime sleep in *neurexin* mutant flies. This conclusion is consistent with previous reports, which show that sleep behavior is closely correlated with synaptic function and transmission^15^,^16^.

As in mammals, *Drosophila* sleep is coordinated by the interaction between sleep-promoting and wake-promoting neurons. Previous studies showed that blocking synaptic output from KCs can either increase or decrease sleep depending on the GAL4 driver used^21^,^22^,^36^. In the present study, we used anatomically restricted GAL4 lines to demonstrate that blocking synaptic output from αβ_sc_ neurons resulted in reduced nighttime sleep, whereas silencing αβ_p_ neurons did not significantly affect nighttime sleep. Consistent with previous observations^22^,^36^,^43^, activation of αβ neurons resulted in normal nighttime sleep. Furthermore, we showed that down-regulation of ACh signaling from αβ_s_ neurons inhibited nighttime sleep, whereas depletion of sNPF from αβ_sc_ neurons decreased nighttime sleep. The αβ_sp_ neurons have been identified as putative cholinergic neurons^42^,^49^, and down-regulation of ACh signaling inhibits nighttime sleep^42^. In addition, sNPF is highly expressed in MBs, and *sNPF* mutant flies display reduced sleep, indicating that sNPF plays an essential role to promote sleep in *Drosophila*^50^,^51^.

Our study finds that different αβ neuron populations release variant neurotransmitters and/or neuropeptides to promote nighttime sleep. The different sleep-promoting roles of αβ_c_ and αβ_s_ neurons might be attributable to the anatomy of neural circuits within the MB lobes. Each MB contains ~2,000 KCs that extend their axonal fibers to relevant lobes and synapse with a relatively small number of MB output neurons (MBONs)^26^,^30^,^52^. Thus, the convergence of a large number of KCs onto a small number of MBONs may integrate signals from both sleep-promoting and wake-promoting neural circuits. MB-M8 dopamine neurons arborize throughout the β_s_ and β_c_ regions and are positively reinforcing during odor stimulation, whereas aversive-reinforcing MB-M3 and MB-MP1 dopamine neurons preferentially arborize in the αβ_s_ layer and exhibit no or much weaker αβ_c_ innervation^49^. A recent report revealed that activation of different MBON populations either suppresses or promotes sleep^26^,^27^. However, the process of signaling integration in different αβ-subset neurons under physiological conditions requires further investigation.

## Methods

### Fly stocks and genetics

Flies were maintained on standard medium at 25 °C, 60–80% relative humidity, with a 12 h light/dark cycle unless otherwise stated. The wild-type flies used in this study were *w*^*1118*^, except for those in Fig. 6A that were *Canton-S* flies. The *α-neurexin (CG7050*) null mutant allele *nrx*^*273*^ was obtained from Dr. Manzoor A. Bhat’s lab^10^; it also carries a mutation in the DNA repair gene *mutagen-sensitive 309 (mus309*) located at 86E17. The *α-neurexin* null mutant allele, *nrx*^*Δ83*^ was generated by imprecise excision of the P-element using Δ2–3 as a transposase source according to standard procedures^9^. *P*{*XP*}*nrx*^*d08766*^ was obtained from the Bloomington Stock Center. To delete the extra mutants and standardize the background, all *neurexin* mutants were outcrossed for more than six generations with the *w*^*1118*^ strain. To further eliminate potential genetic background effects, two out-crossed *neurexin* null mutant allele *nrx*^*Δ83/273*^ were combined for use in this study. The full-length neurexin transgene *p[UAS-Neurexin]* was generated as previously described^9^,^53^. The mushroom body split-GAL4 lines MB185B, MB594B, and MB371B were kindly provided by Yoshi Aso. UAS-RNAi lines were ordered from the Tsinghua Fly Center and Vienna Drosophila RNAi Center. Other lines used in this work were obtained from the Bloomington Stock Center.

### Sleep and circadian assays

Sleep assays were performed as described previously^19^. For sleep measurements, 3- to 5-day-old male flies raised in light/dark (LD)-entrained cultures were placed in 65 × 5 mm glass tubes containing 5% sucrose and 2% agar. Locomotor activity was measured for 5–7 days at 25 °C during LD cycles using the Drosophila Activity Monitoring System (DAM2, Trikinetics) in a DigiTherm CircKinetics incubator (Tritech Research). Data were collected in 1-min bins, and a sliding window was applied. Sleep was defined as 5 consecutive minutes of inactivity as described previously^54^. Data were analyzed with pySolo software. For analysis of circadian behavior, locomotor activity was measured using DAM2 monitors (Trikinetics), and activity counts were collected in 30 min bins in LD for 4 days, and then in DD during a 7 day period, and analyzed using ClockLab (Actimetrics). Power is a measure of rhythm amplitude and corresponds to the height of the periodogram peak above the significance line^55^.

For temperature-shift experiments, flies expressing *UAS-dTrpA1* were raised at 21 °C throughout their development, and dTrpA1 was activated at 28 °C. Flies expressing *Tubulin-GAL80*^*ts*^ were reared at 18 °C throughout their development, and adults were entrained at 30 °C for 6 hours every day for 2 days. Then, sleep was measured. To exclude the potential role of elevated temperature in sleep, sleep data collected at the elevated temperature were compared with those collected at the normal temperature.

Sleep deprivation experiments were performed as described previously^56^,^57^. Briefly, male flies were subjected to sleep deprivation using the mechanical Sleep Nullifying Apparatus (SNAP) method while housed in TriKinetics DAM2 systems. Cumulative difference plots were calculated for each individual fly by comparing the percentage of sleep lost during overnight sleep deprivation to that of the immediately preceding unperturbed night. Individual sleep rebound was quantified every hour for 24 h by dividing the cumulative amount of sleep regained by the total amount of sleep lost during deprivation. We found that 9 h of sleep deprivation was sufficient to cause a 70% loss of sleep in *w*^*1118*^ flies and more than 60 min of sleep loss in some mutant flies. To exclude the flies escaping from mechanical stimulations, individual flies were excluded from rebound analysis if 12 h of sleep deprivation was less than 70% effective in control flies or if mutant flies lost less than 60 min of sleep^57^. Statistical significance was assessed by two-way repeated-measures ANOVA.

### Antibodies

The anti-neurexin antibody was generated as described previously^9^,^53^. Briefly, purified 6 × His fusion fragment (amino acids 1,534–1,690) of neurexin protein was used as antigen. An affinity column was created by coupling GST-neurexin fusion protein to CNBr-activated Sepharose 4B, and was used to purify the antibody. Other antibodies were obtained from Developmental Studies Hybridoma Bank (Tubulin, Fas II, and nc82).

### Western blots

Fly heads were homogenized in SDS sample buffer. The proteins were fractionated by SDS-PAGE and transferred to polyvinylidene fluoride (PVDF) membranes (Pall) in Tris-glycine buffer. After blocking, the membranes were probed with anti-neurexin (1:200) or anti-tubulin (1:1,000) antibody at room temperature for 2 h. After three washes with phosphate-buffered saline containing Tween, the membranes were probed with either anti-rabbit or anti-mouse IgG-peroxidase conjugate (GE Healthcare) at room temperature for 2 h. The probe signals were detected using enhanced chemiluminescence reagents (Amersham Biosciences).

### Immunostaining

After dissection and fixation, fly heads were stained with anti-GFP (1:100), anti-FasII (1:100, Developmental Studies Hybridoma Bank), and anti-Brp (1:100, nc82, Developmental Studies Hybridoma Bank) antibodies. Samples were imaged on an LSM 710 confocal microscope (Zeiss). For Cac-GFP live imaging, dissected adult heads were mounted on the slides and the images were acquired from live α lobes using an LSM 710 confocal microscope (Zeiss).

### *In vivo* calcium imaging

*In vivo* GCaMP imaging experiments were performed on 3- to 7-day-old male adult flies. GCaMP6.0 and mCD8-RFP were expressed in αβ-subset neurons under *c739-GAL4* control; mCD8-RFP was used as a control. Adult flies were entrained under a 12-h LD cycle, and the fly brains were rapidly dissected at ZT21 under a light intensity of 400 lux for <90 s. After resting in complete darkness for more than 30 min, GCaMP6.0 and mCD8-RFP signals from the αβ lobe were imaged at ZT22. The GFP/RFP ratio is presented.

*In vivo* Cam2.1 imaging experiments were performed as described previously^58^. Briefly, fluorescence signals were monitored on an LSM 710 confocal microscope (Zeiss). Each experiment was performed with more than 8 flies, and averaged responses were presented. Data acquisition and evaluation were performed as described previously^58^.

### Statistical analysis

Data are presented as mean ± SEM. Two-tailed Student’s *t*-tests were used to compare genotypes for statistical analysis of sleep parameters with normal distributions. Mann-Whitney U-tests were used to analyze sleep episode length, which did not have normal distributions. Signal intensities of western blots and calcium images were measured with ImageJ software (National Institute of Health, USA), and data from eight independent experiments were averaged. Statistical significance was set as *p* < 0.05, *p* < 0.01, *p* < 0.001, or no significance.

## Additional Information

**How to cite this article**: Tong, H. *et al*. Neurexin regulates nighttime sleep by modulating synaptic transmission. *Sci. Rep.* **6**, 38246; doi: 10.1038/srep38246 (2016).

**Publisher’s note:** Springer Nature remains neutral with regard to jurisdictional claims in published maps and institutional affiliations.

## Supplementary Material

Supplementary Data
