# Supplementary material for: Neurexin regulates nighttime sleep by modulating synaptic transmission
Source: Sci Rep. 2016 Dec 1;6:38246. doi: 10.1038/srep38246 (PMC5131284; doi:10.1038/srep38246)
Supplement: Supplementary Data [file srep38246-s1.pdf]

Neurexin regulates nighttime sleep by modulating synaptic transmission

Huawei Tong[1], Qian Li[1], Zi-Chao Zhang[1], Yi Li[1], and Junhai Han[1,2,*]

1. Institute of Life Sciences, Key Laboratory of Developmental Genes and Human Diseases, Southeast University, Nanjing 210096, China

2. Co-Innovation Center of Neuroregeneration, Nantong University, Nantong, JS 226001, China

* Corresponding author: Junhai Han, Institute of Life Sciences, Southeast University, 2 Sipailou Road, Nanjing, 210096, China. Tel: +86-25-83790962; Fax: +86-25-83790962; E-mail: junhaihan@seu.edu.cn

Supplementary Figures and Figure Legends

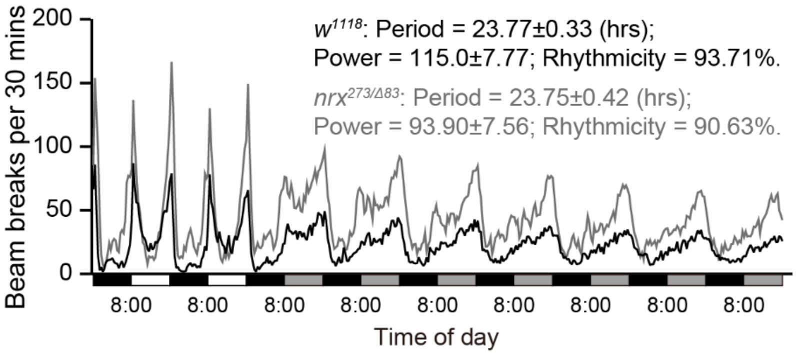

Figure S1. The $nrx^{\Delta 83/273}$ mutants show normal circadian locomotor rhythmicity.

White and grey areas indicate light and dark conditions, respectively. Quantification of circadian periods is presented in the top panel (*n*=32). Power is a measure of rhythm amplitude and corresponds to the height of the periodogram peak above the significance line.

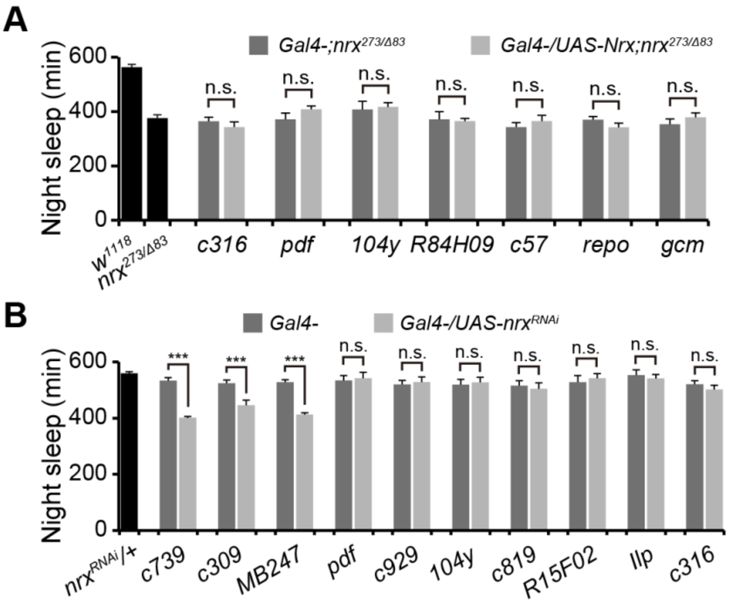

Figure S2. Neurexin expressed in MB neurons is essential for nighttime sleep.

(A) Total nighttime sleep in flies with rescued neurexin expression using anatomically restricted GAL4 drivers. All rescue experiments were performed in the *nrx*$^{273/\Delta 83}$ background, and flies carry one copy of the indicated drivers (*n*=32). **(B)** Total nighttime sleep in flies with neurexin depletion using *UAS-nrx*^{RNAi} driven by anatomically restricted GAL4 drivers. A single copy of the driver was used for each GAL4 line (*n*=32).

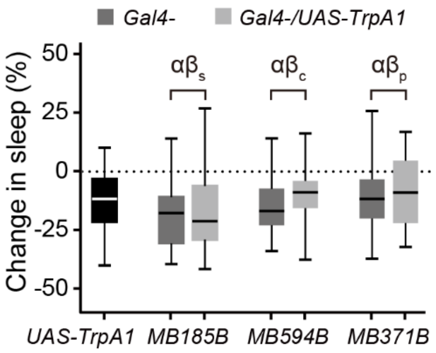

Figure S3. Activation of αβ neurons leads to normal nighttime sleep.

Quantification of sleep changes induced by dTrpA1 activation under each of the indicated split-GAL4 driver lines (*n*=32). The horizontal line dividing the box is the median.

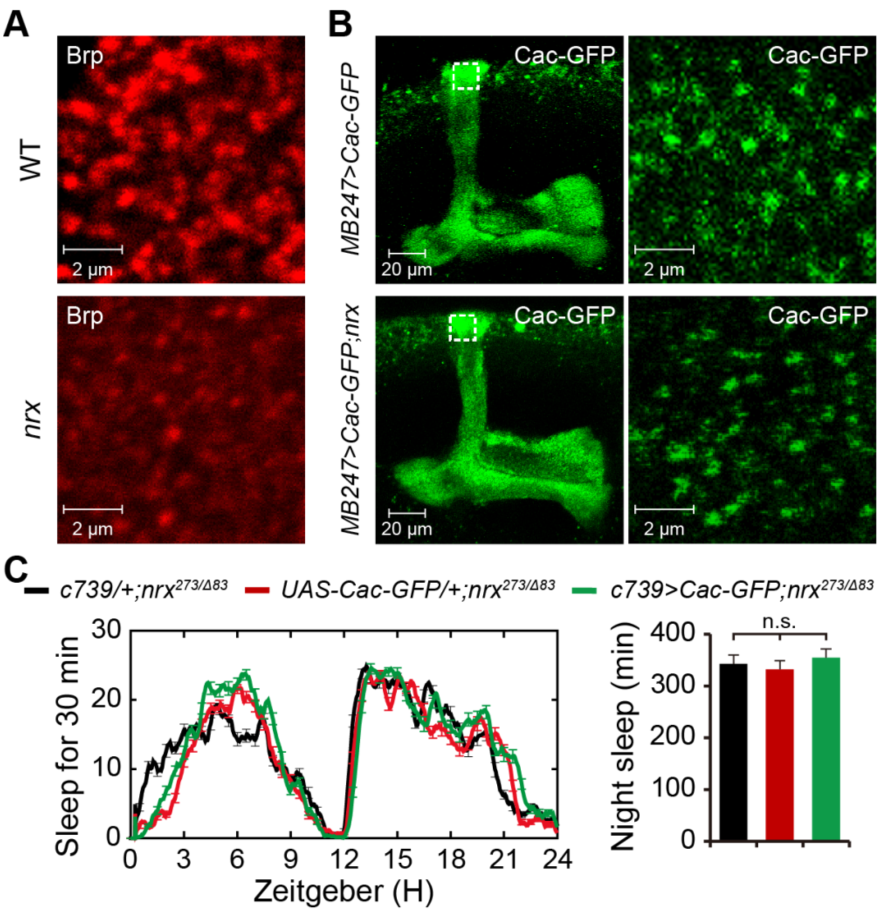

Figure S4. The *neurexin* mutant α-lobes show normal Cac channel levels and distributions.

(A) Brp staining in α-lobes of wild-type and *neurexin* mutants. Note that the level of Brp is reduced in *neurexin* mutants. Scale bars=2 μm. **(B)** Confocal images of α-lobes expressing Cac-GFP in MB neurons in wild-type and *neurexin* mutants. No significant changes in the signal intensity between the two genotypes were found (wild type, 100±5.2, *n*=6; $nrx^{273/\Delta83}$, 92.9±4.3, *n*=7). **(C)** Average sleep profiles for $UAS\text{-}Cac\text{-}GFP/c739\text{-}GAL4;nrx^{273/\Delta83}$ and control flies, plotted as a 30 min moving average, and quantification of total nighttime sleep for each genotype is presented in the right panel (*n*=32).

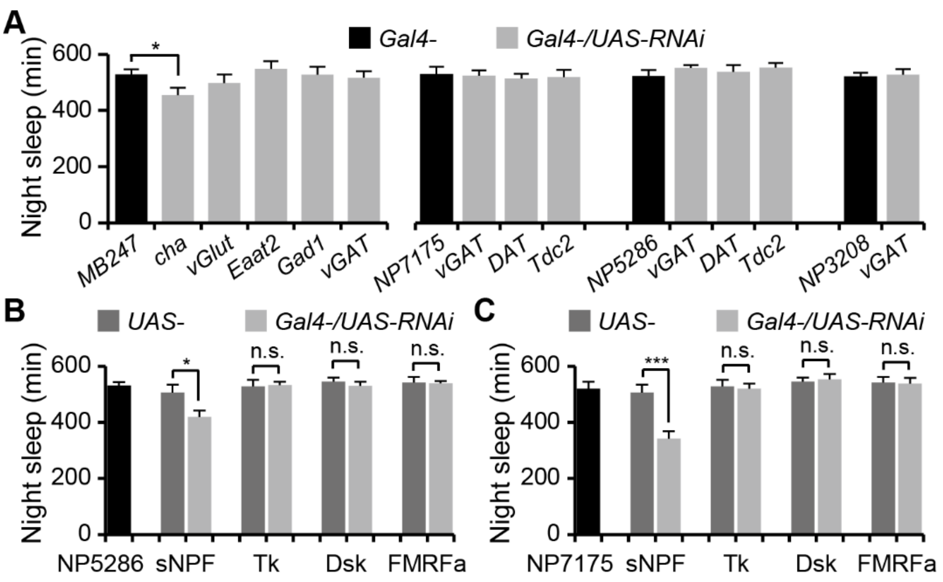

Figure S5. Depletion of acetylcholine or short neuropeptide F (sNPF) in mushroom body neurons reduces nighttime sleep.

(A) Quantification of total nighttime sleep in flies subjected to RNAi for neurotransmitter-related genes and control flies (*n*=24). **(B-C)** Quantification of total nighttime sleep time in flies subjected to RNAi for neuropeptide and control flies; both *NP5286-GAL4* **(B)** and *NP7175-GAL4* **(C)** were used in RNAi experiments (*n*=16).
